# The Rare Case of Esophageal Cancer Presenting With Hematemesis in a 29-Year-Old Adult

**DOI:** 10.7759/cureus.39881

**Published:** 2023-06-02

**Authors:** Rabira R Dufera, Abdallah Osman, Ikenna Nnamani, Oluwaremilekun Tolu-Akinnawo, Duane Smoot

**Affiliations:** 1 Internal Medicine, Meharry Medical College, Nashville, USA; 2 Internal Medicine, Meharry Medical College, Nashville , USA; 3 Gastroenterology and Hepatology, Meharry Medical College, Nashville, USA

**Keywords:** neo-adjuvant chemotherapy, barret esophagus, hematemesis, esophagus adenocarcinoma, gastro-esophageal disease, ec- esophageal cancer, esophageal squamous cell carcinoma (scc)

## Abstract

Esophageal cancer is a disease with high mortality. This is mainly due to late presentations with nonspecific symptoms. Despite advances in surgery and chemoradiotherapy, it is the eighth most common cancer but the sixth deadliest. It is reportedly common in older patients but rare in young ones. In this case report, we present a 29-year-old male patient with no prior medical condition who presented with hematemesis to the emergency unit and was found to have esophageal cancer with the biopsy. Not only is esophageal cancer rare in young adults, but hematemesis is a rare symptom in esophageal cancer patients.

## Introduction

Esophageal cancer is a devastating disease. The global incidence of esophageal cancer varies geographically. High-prevalence areas include Asia and southern and eastern Africa. The two types of esophageal carcinoma are squamous cell carcinoma and adenocarcinoma. Worldwide, squamous cell carcinoma makes up about 90% of all esophageal cancers; however, the incidence has been decreasing, whereas the incidence of adenocarcinoma has been rising. Esophageal cancer occurs in the fifth to seventh decades of life and is three to four times more common in men. About 16,000 new cases occur annually in the United States, with 15,000 deaths in the same year. The overall five-year survival rate ranges from 15% to 25%, depending on the cancer stage at initial presentation [1].

## Case presentation

**Figure 1:**
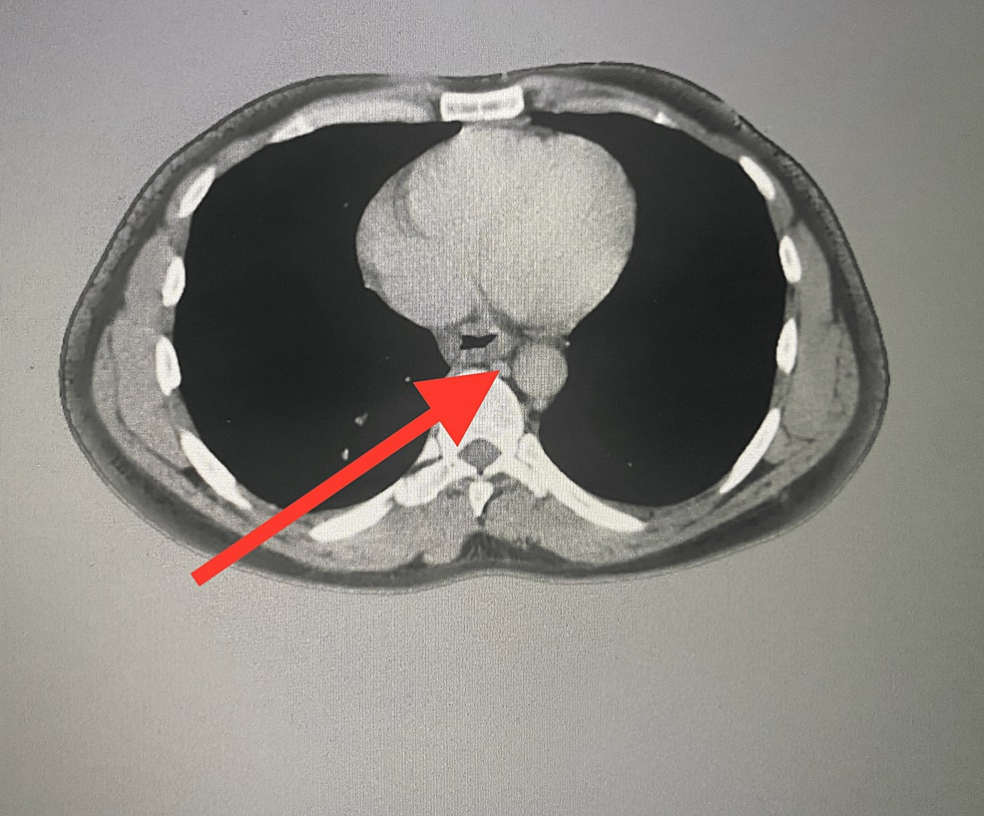
Computed tomography of the chest with contrast showed luminal narrowing of distal esophagus as shown with the arrow.

The patient is a 29-year-old male with no prior medical conditions who presented to the emergency department on account of hematemesis of one-day duration. He denied any prior history of hematemesis or family history of esophageal cancer but reported an on-and-off history of reflux symptoms for the last year, especially when eating spicy foods, but mentioned that those symptoms go away by themselves and thus did not seek any medical help. Of note, the patient also mentioned that he lost about six pounds over the last six months prior to the presentation, which he also attributed to stress at work. On careful questioning, the patient mentioned a preference for soft diets, which he prefers more as they are easier to chew and swallow. He mentioned that he had to drink more water to help with swallowing, especially after eating steaks for the past year. He did not have any additional gastroenterological disorders or a significant family history of gastroenterological malignancies. His social history is significant because he smoked a pack of cigarettes over the last 5 years. He denied drinking alcohol. On admission, he was hemodynamically stable, and there was no active bleeding. Initial laboratory work-up, including complete blood cell counts, liver function tests, and coagulation studies, was normal. Computed tomography of the chest and abdomen with intravenous contrast showed circumferential thickening of the distal esophagus, which may be secondary to esophagitis versus neoplastic etiology (Figure 1).

Esophagogastroduodenoscopy (EGD) was done as the patient also had unintentional weight loss and dysphagia. The finding was significant for an exophytic, noncircumferential mass on the distal esophagus extending to the gastro-esophageal junction. The mass was biopsied, and the histology result showed squamous cell cancer of the esophagus. The mass measured about 6 mm in thickness. There was sonographic evidence of invasion into the submucosa. The multidisciplinary team then evaluated the patient. Staging workups using positron emission tomography (PET) scans and computed tomography (CT) scans of the abdomen/pelvis revealed no evidence of distant metastasis. He was diagnosed with early-stage squamous cell cancer (cT1bN0M0) based on the American Joint Committee on Cancer Staging System (AJCC) and referred to hemato-oncology for further management.

## Discussion

Esophageal cancer is among the cancers on the rise globally. The cancer has increased recently. Variation in incidence is common with geographical location [1]. Genetics, ethnicity, and lifestyle have contributed to variations in incidence. Especially squamous cell cancer has a high prevalence in East Africa, South Africa, South Europe, and East Asia. Nonetheless, the incidence rate is lower in North America and other parts of Europe [2]. For esophageal adenocarcinoma, the only known precursor lesion is Barrett's esophagus. Barrett's esophagus increases the risk of having esophageal adenocarcinoma about 30-40 times [3]. Unfortunately, only 5% of esophageal adenocarcinomas have a precancer diagnosis of Barrett's esophagus, warranting the identification of additional risk factors [4].

Pathology

Adenocarcinomas are most common in the lower third of the esophagus and at the gastro-esophageal junction, with the incidence rising sharply over the last few decades in the US. Squamous cell carcinomas are more common in the upper and middle esophagus.

Risk factors

Esophageal cancer is more common in the fifth to seventh decades of life. It is three to four times more common in males than females. Esophageal adenocarcinoma is more common in white males than in Hispanics or black people [5]. However, esophageal squamous cell cancer (ESCC) accounts for 87% of all esophageal cancers in black people but only accounts for about 45% in whites [5]. Other risk factors for developing esophageal cancer include smoking, alcohol consumption, and gastroesophageal reflux disease. Ten percent of patients with gastro-esophageal disease develop Barrett's esophagus [6]. Genetic mutations play a key role in the development of esophageal cancer. For this particular case report, there was no genetic testing done, and there was no family history of gastrointestinal carcinoma. For example, more than 80% of ESCCs are associated with a mutation of the tumor suppressor gene TP53 [7]. Moreover, 78.6% of ESCC patients have mutations and/or amplifications in factors downstream of the epidermal growth factor receptor (EGFR), including a receptor tyrosine kinase, rat sarcoma (RAS), and Ak strain transforming (AKT) pathways [7]. In esophageal adenocarcinoma patients, the expression of B cell translocation gene 3 (BTG3) was low in tumor tissues when compared to adjacent normal tissues. The level of BTG3 expression was associated with lymph node metastasis and tumor staging [8].

Clinical features of esophageal cancers

A small percentage of patients have asymptomatic tumors detected during endoscopy for unrelated causes or surveillance for Barrett's esophagus. However, most patients present with dysphagia, initially with solids when the esophageal lumen is 13 mm or less but progressing to liquids as the tumor grows and the lumen narrows. The diagnosis may be late because patients adjust their dietary intake to avoid foods that cause dysphagia. Other less common presentations include cough, hoarseness due to recurrent laryngeal nerve involvement, palpable cervical lymph nodes, and iron deficiency anemia from chronic gastrointestinal blood loss. Upper gastrointestinal bleeding (UGIB) accounts for 5.7% of the total emergency department visits of esophageal cancer patients [9]. UGIB usually presents as melena or hematemesis and is a life-threatening condition requiring urgent treatment. Most importantly, the cause of UGIB in esophageal cancer, as in this case report, is tumor ulcer. When compared to non-tumor ulcers or variceal bleeding, esophageal cancer may seem to be a rare cause of UGIB. However, a previous study showed that in gastrointestinal cancer patients, 20% of UGIB was due to tumor bleeding, which is notably higher than in patients without a cancer history [10].

Diagnostic testing

The diagnosis is established through upper endoscopy with a biopsy. The staging workup includes CT of the chest and abdomen, with or without the addition of a PET scan to determine the presence of distant metastases. For patients without distant metastases, endoscopic ultrasonography (EUS) should be done to define tumor depth and lymph node status for staging and treatment planning. Tumors located above the carina increase the risk of tracheoesophageal (TE) fistula formation and should be evaluated with bronchoscopy. Patients with TE fistulas often present with a postprandial cough and aspiration pneumonia.

Staging

The tumor node metastasis (TNM) classification is similar between squamous and adenocarcinomas, including Tis (carcinoma in situ), T1 (invades lamina propria, muscularis mucosa, or submucosa), T2 (invades muscularis propria), T3 (invades adventitia), T4 (invades adjacent structures), N0 (no lymph node involvement), N1 (1-2 regional lymph nodes), N2 (3-6 regional lymph nodes), N3 (7 or more lymph nodes), M0 (no metastases), and M1 (distant metastases). Staging varies slightly by histology, but in general: stage I (T1N0), stage II-III (T2-3 and/or N1), stage IVA (T4 or N2-3), and stage IVB (M1).

Treatment of esophageal cancers

For stage I esophageal cancer, endomucosal or surgical resection is sufficient. For stages I-III (locally advanced, resectable), the standard therapy is concurrent neoadjuvant chemoradiation with a platinum-based regimen followed by surgical resection [11]. Patients who have residual disease on postoperative pathology may benefit from adjuvant nivolumab [12]. For stage IVA (locally advanced, unresectable), the standard of care is definitive concurrent chemoradiation with a platinum-based regimen [13]. For stage IVB or metastatic disease, the treatment is with combination chemotherapy regimens including fluoropyrimidines (5-FU or capecitabine), platinum agents (cisplatin or oxaliplatin), taxanes (docetaxel or paclitaxel), and irinotecan. Patients with adenocarcinoma and HER2+ tumors should receive trastuzumab in combination with first-line chemotherapy. Patients with PD-L1 CPS ≥5 may benefit from the addition of pembrolizumab to initial chemotherapy.

## Conclusions

Esophageal cancer remains a significant cause of all cancer-related deaths worldwide. Strikingly, the incidence is on the rise in the western hemisphere, with more presentation in young adults, as in this case. As esophageal cancer has a potential for late presentation, it is wise to have a high degree of suspicion, evaluate the patient for esophageal cancer, and initiate prompt treatment. One way of evaluating for esophageal cancer prior to presentation with hematemesis, as in this case report, is to do an upper gastrointestinal endoscopy given his history of preferences for soft diets and reflux symptoms for one year, as well as his weight loss, although he attributes it to stress at work.
